# Optimizing adolescent inguinal hernia repair: a comprehensive robotic-assisted laparoscopic approach

**DOI:** 10.1007/s10029-026-03889-z

**Published:** 2026-10-26

**Authors:** J. Benjamin Pitt, Ashley C. Dodd, Alison Lehane, Micah Holloway, Edward Gong, Guillermo Ares, Timothy B. Lautz, Seth D. Goldstein

**Affiliations:** 1 Division of Pediatric Surgery, Department of Surgery, Northwestern University Feinberg School of Medicine, Ann and Robert H. Lurie Children’s Hospital of Chicago, 255 E Chicago Avenue, Chicago, IL 60611 USA; 2 Division of Urology, Department of Surgery, Northwestern University Feinberg School of Medicine, Ann and Robert H. Lurie Children’s Hospital of Chicago, Chicago, IL 60611 USA

**Keywords:** Minimally invasive, Robotic-assisted, Laparoscopic inguinal hernia repair, Pediatric surgery

## Abstract

**Purpose:**

Consensus guidelines for the management of adolescent inguinal hernia repair remain lacking. While high ligation remains common among pediatric surgeons, mesh-based floor reinforcement is selectively used by some surgeons for adolescents with larger or more complex hernias. Consequently, significant variability exists in the surgical management of adolescents with inguinal hernias. In this study, we describe an institutional algorithm that utilizes an individualized approach to adolescent inguinal hernia repair.

**Methods:**

We conducted a retrospective analysis of adolescents aged 12–18 years who underwent laparoscopic inguinal hernia repair (LIHR) or robotic-assisted transabdominal preperitoneal (rTAPP) inguinal hernia repair with mesh over a four-year period, following a standardized decision-making algorithm. If on initial diagnostic laparoscopy there was a processus vaginalis > 2 cm, long hernia sac with scrotal component, or direct component, we proceeded with rTAPP. Otherwise, LIHR was performed.

**Results:**

44 adolescents underwent inguinal hernia repair using this algorithm. For LIHR, median (IQR) operative time, age, and weight were 20 min (13–38), 15 years (14–16), and 59 kg (53–66), respectively. For rTAPP, these were 115 min (105–133), 16 years (15–17), and 67 kg (58–77), respectively. Only operative time was statistically significant between the two cohorts. There were neither conversions to open, postoperative admissions, nor instances of recurrence to date.

**Conclusion:**

Our institution successfully implemented a tailored algorithm for adolescent hernia repair based on age and hernia characteristics. Mesh was well tolerated without additional morbidity and no recurrences during available follow-up. This highlights the safety, effectiveness, and relevance of a customized algorithm in this unique population.

## Introduction

Contemporary minimally invasive approaches to adolescent inguinal hernia repair vary among pediatric surgeons [1–9]. The most widely practiced is the laparoscopic inguinal hernia repair (LIHR). This offers high ligation of an indirect hernia alone, without reinforcement of the inguinal ring or floor [4, 10]. This is a commonly performed operation by pediatric surgeons on infants and younger children with high rates of success and adoption; however, there are factors that may render the LIHR technique insufficient for many adolescents. Namely, large patulous indirect defects with a wide internal inguinal ring, extension into the scrotum, or direct components of the hernia which can lead to recurrence with high ligation alone [11]. Accordingly, some pediatric surgeons have advocated for treating large adolescent hernias with floor reinforcement, which in a minimally-invasive paradigm involves mesh via robotic-assisted transabdominal preperitoneal (rTAPP) repair [12, 13].

Generally, the utilization of minimally invasive approaches for pediatric hernias has been increasing over time, has demonstrated safety and efficacy across various procedures, and is used by centers worldwide for repair of inguinal hernias [14–18]. Studies have shown that oftentimes the approach to inguinal hernia repair is more dependent upon surgeon preference or training than patient or hernia characteristics [1, 4]. While some centers have reported on their experiences with various minimally invasive approaches to adolescent inguinal hernia repair, the absence of a standardized algorithm to guide surgical decision-making toward the most efficacious repair remains a notable gap in the literature [19, 20].

As the utilization of laparoscopic and robotic-assisted laparoscopic techniques for older children evolves, we have developed an institutional algorithm that guides a personalized approach to offer available options for adolescent minimally invasive inguinal hernia repair. In this study, we present our experience utilizing this algorithm as a tertiary care center specializing in the surgical care of pediatric patients. Through retrospective evaluation of all adolescent patients who underwent minimally invasive inguinal hernia repair at our institution in accordance with our algorithm, we aim to provide support for a standardization of the surgical approach to minimally invasive adolescent inguinal hernia repair.

## Methods

### Study population

Institutional review board approval was obtained from Ann and Robert H. Lurie Children’s Hospital of Chicago (IRB 2024–6716). This was a retrospective evaluation of all adolescents ages 12 to 18 years old who underwent minimally invasive inguinal hernia repair at our institution from March 2022 to April 2026 adhering to a standard decision-making algorithm that was implemented in 2022.

### Data source and collection

Patients meeting the inclusion criteria who underwent minimally invasive inguinal hernia repair during the study period were identified through chart review. Patient characteristics including age, sex, weight, and race/ethnicity, as well as surgical details including approach (LIHR versus rTAPP), laterality, and operative time, were extracted from the medical record and operative notes.

### Algorithm design and operative approach

**Fig. 1:**
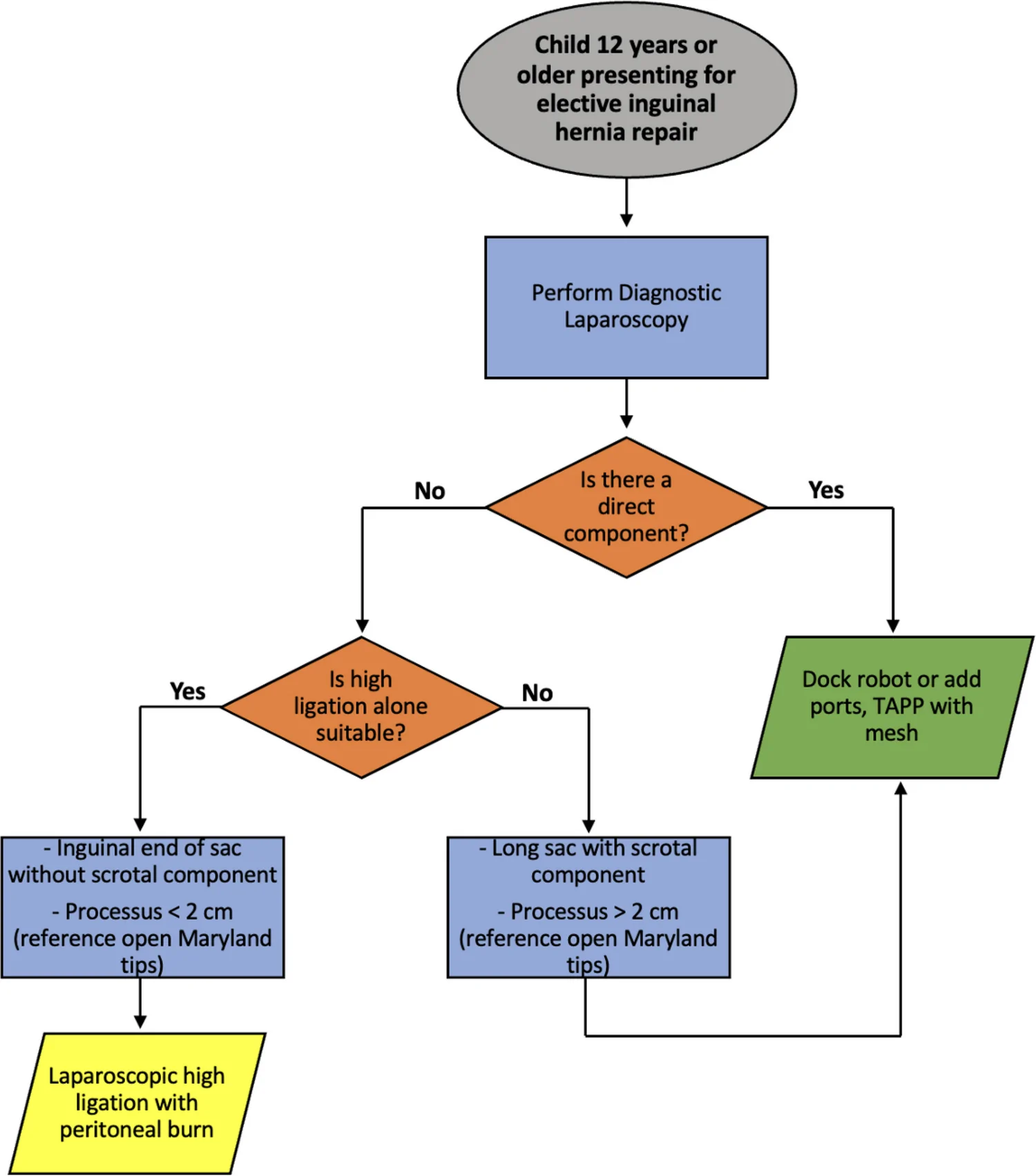
Adolescent inguinal hernia repair algorithm. Robotic-assisted laparoscopic inguinal hernia repair algorithm for patients 12 years and older with decision points (orange diamonds) regarding type of inguinal hernia and the appropriateness of high ligation using the LIHR technique

Our algorithm is designed to guide surgeons’ intraoperative decision-making in the setting of minimally invasive adolescent inguinal hernia repair (Fig. 1). The two techniques utilized in the algorithm are LIHR and rTAPP, both of which are described as adequate techniques in the repair of inguinal hernia; however, the optimal approach relies on several deciding factors related to hernia characteristics that are outlined in our algorithm.

**Fig. 2:**
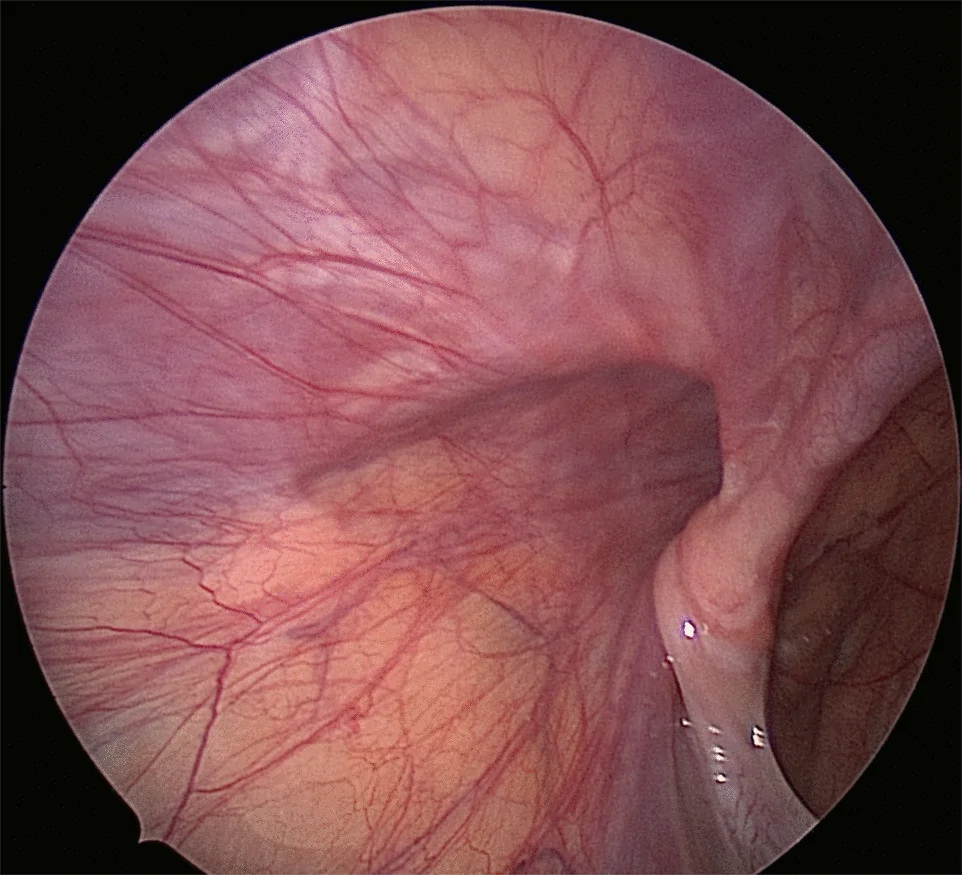
Conditions for high ligation. Diagnostic laparoscopy demonstrating conditions for high ligation LIHR: small indirect inguinal hernia (< 2 cm) without long scrotal component

All cases begin with diagnostic laparoscopy for evaluation of the inguinal hernia with the placement of a 5 mm laparoscopic trocar via Veress technique, and a 5 mm laparoscopic Maryland grasper is inserted in a stab fashion without a port. The decision tree depicted in Fig. 1 then commences. If upon initial evaluation there is not a direct component, scrotal extension, or defect greater than the fully spread grasper (Fig. 2), standard laparoscopic inguinal hernia repair is performed using the existing instrumentation, according to the surgeon’s preferred technique.

**Fig. 3:**
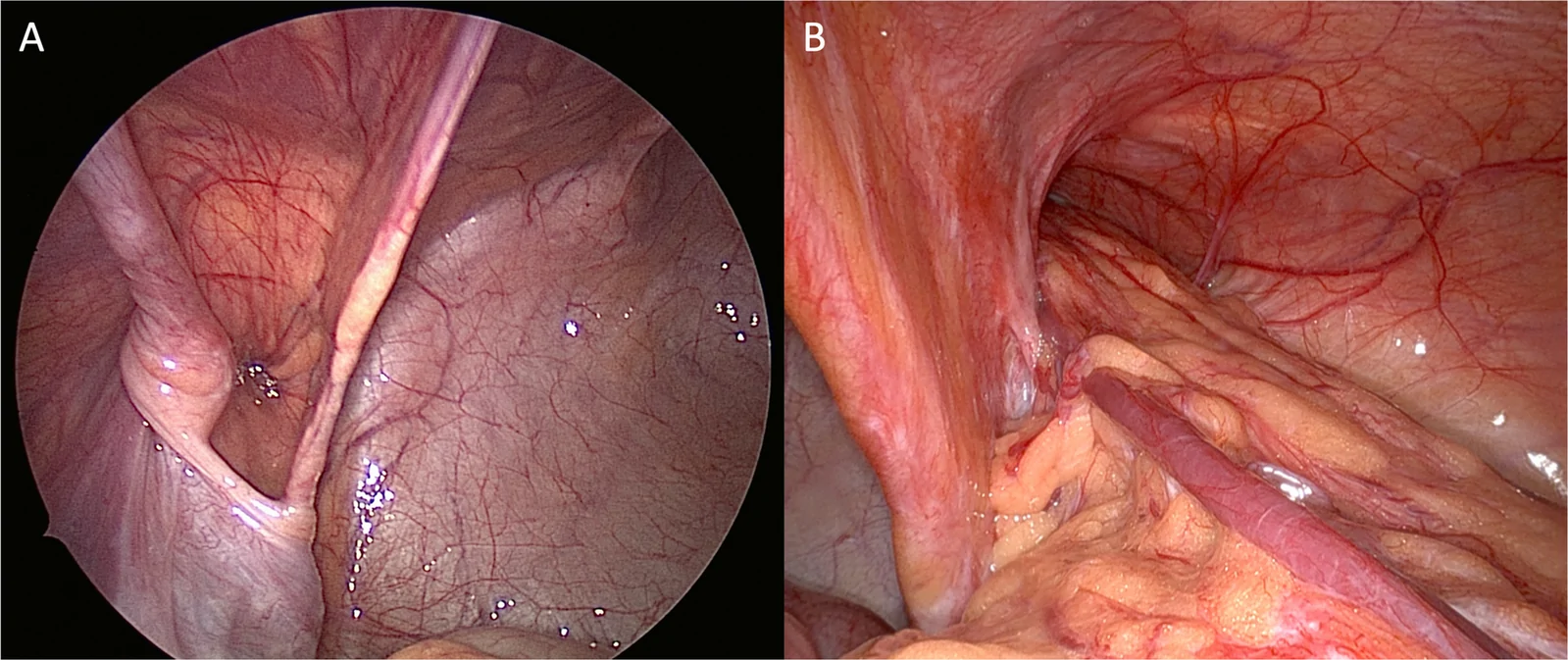
Conditions for rTAPP. Diagnostic laparoscopy demonstrating conditions met for rTAPP: **A** left-sided direct hernia defect; **B** large right indirect inguinal hernia with a long hernia sac, scrotal component, and a processus vaginalis greater than 2 cm

**Fig. 4:**
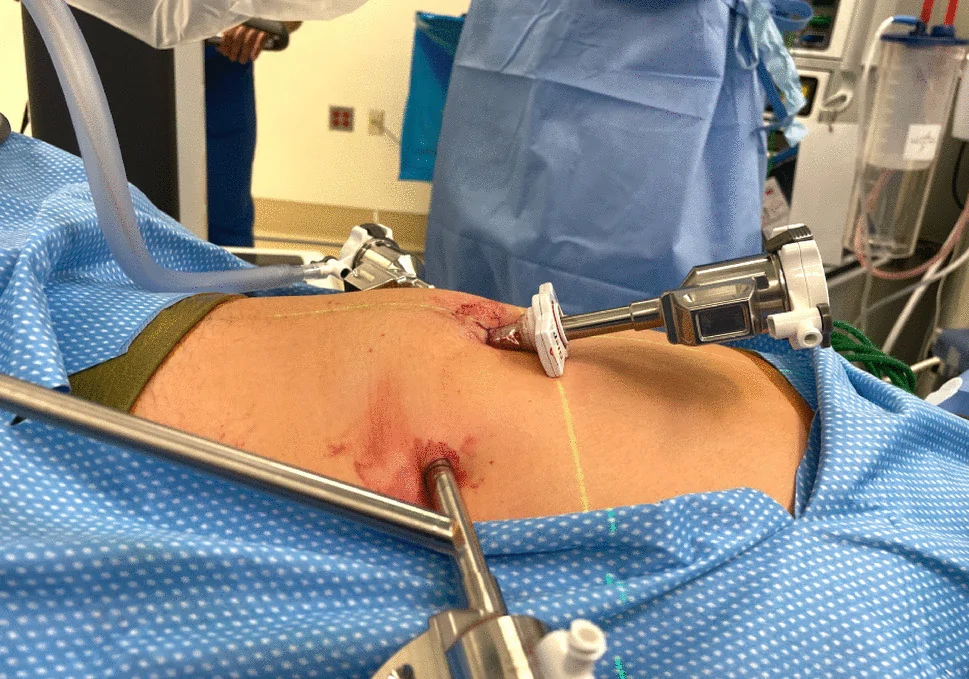
Robotic trocar configuration. Robotic port placement comprising two lateral robotic trocars and an umbilical trocar through the Veress sheath used for initial laparoscopy

**Fig. 5:**
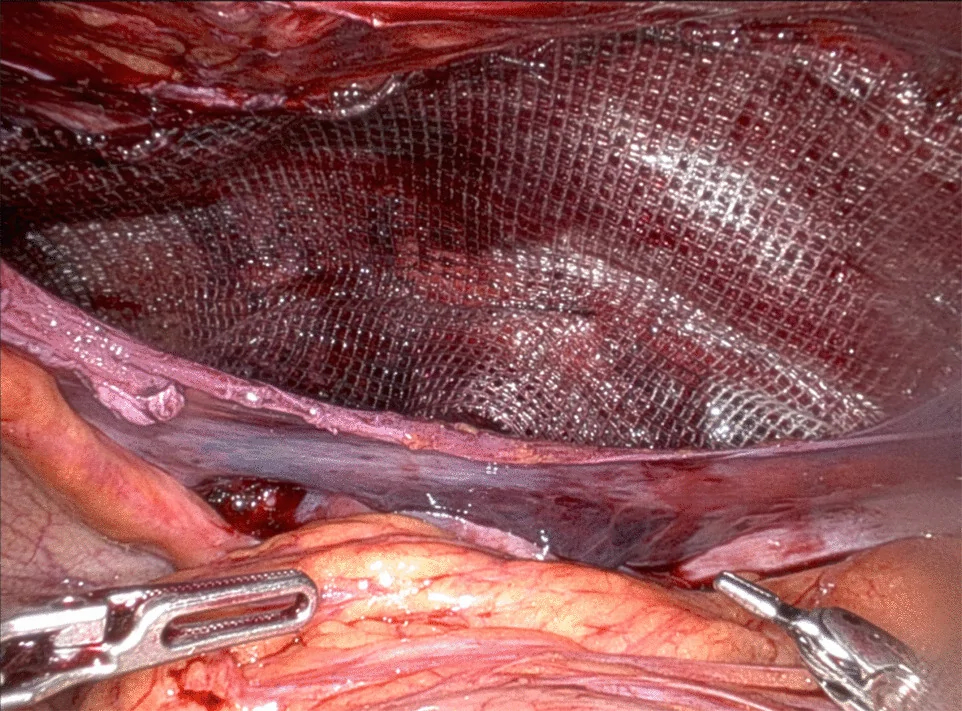
rTAPP mesh placement. Synthetic mesh placed over the direct and indirect spaces in the preperitoneal space during rTAPP

If any of the aforementioned conditions are present (Fig. 3), the decision to perform a three-port rTAPP with mesh is made. Two bilateral 8 mm robotic trocars are placed under direct visualization, including one at the site of the existing Maryland grasper incision. The 5 mm port is withdrawn, leaving the soft tissue sheath in place, and exchanged for a third 8 mm trocar under direct visualization with the camera via the lateral port (Fig. 4). After the robotic system is docked, any herniated viscera is reduced and the peritoneum is incised around the level of the arcuate line to create and develop a peritoneal flap. This is extended inferiorly to include hernia sac, while visualizing spermatic vessels and vas deferens and keeping these structures out of harm’s way. When this has been successfully completed, a mesh is placed over the direct and indirect spaces (Fig. 5). Finally, the peritoneum is closed in a running fashion [5]. All patients are discharged home on the day of operation without opioid prescription and instructed to follow-up in 2–3 weeks for postoperative evaluation.

### Data analysis

Descriptive statistics were used to identify age, sex, race/ethnicity, weight, laterality, and operative time of patients undergoing LIHR and rTAPP. Statistical significance of these characteristics between techniques was evaluated using Mann–Whitney U tests for age, weight, and operative time, Fisher’s exact tests for sex and laterality, and Chi-squared test for race/ethnicity.

## Results

From March 2022 to April 2026, 44 adolescents underwent elective inguinal hernia repair guided by our operative decision-making algorithm (Table 1). Of these patients, 15 (34%) underwent LIHR while 29 (66%) underwent rTAPP. Most patients were white, non-Hispanic (*n* = 25, 57%) males (*n* = 41, 93%). Median operative time among all patients was 87 min. To date, there have been no intraoperative complications, conversions to open repair, or postoperative admissions.

**Table 1:** Patient Demographics and Surgical Characteristics. Demographic and surgical characteristics of all adolescents undergoing high ligation of the hernia sac via laparoscopic inguinal hernia repair (LIHR) and robotic-assisted transabdominal preperitoneal (rTAPP) inguinal hernia repair with mesh

**Characteristic**	**LIHR** (*n* = 15)	rTAPP* (*n* = 29)	***p*-value**
Age (y), Median (IQR)	15 [14-16]	16 [15-17]	0.183
Sex, N (%)			0.218
Male	13 (87%)	28 (97%)	
Female	2 (13%)	1 (3%)	
Weight (kg), Median (IQR)	59 [53–66]	67 [58–77]	0.075
Race/Ethnicity			0.716
White, Non-Hispanic	9 (60%)	16 (55%)	
Hispanic	4 (26%)	5 (17%)	
Black	1 (7%)	4 (14%)	
Other	1 (7%)	4 (14%)	
Laterality			0.264
Unilateral	13 (87%)	28 (97%)	
Bilateral	2 (13%)	1 (3%)	
Operative Time (min), Median (IQR)	20 [13–38]	115 [105–133]	< 0.001

*LIHR* Laparoscopic inguinal hernia repair, *rTAPP* robotic-assisted transabdominal preperitoneal inguinal hernia repair, *N* Number, *y* years, *IQR* Interquartile range, *kg* kilograms, *min* minutes

*One patient underwent both LIHR and rTAPP during the same operation and is included in the rTAPP group as bilateral repair

Two patients underwent bilateral LIHR. One patient simultaneously underwent an rTAPP for a larger symptomatic hernia and a LIHR for a smaller contralateral hernia and was classified with the rTAPP cohort.

### Laparoscopic Inguinal Hernia Repair (LIHR)

15 patients underwent LIHR using the operative decision-making algorithm. Median weight was 59 kg. Most repairs were unilateral (n = 13, 87%). All patients presented for follow-up, none of whom had evidence of hernia recurrence.

### Robotic-Assisted Transabdominal Preperitoneal Repair (rTAPP)

Among 29 patients who underwent rTAPP, median age was 16 years and all but 1 were male. Median weight was 67 kg, but not significantly different from the LIHR group. Median operative time was 115 min. 24 (83%) have been evaluated for follow-up with none demonstrating hernia recurrence. One patient with a history of thrombocytopenia developed a scrotal hematoma which resolved after 10 days without further complication. One patient reported paresthesia in the lateral femoral cutaneous region, which resolved with time, and lastly, one patient reported UTI following surgery that resolved with antibiotics.

When comparing patient and operative characteristics between operative approaches, there was no statistically significant difference between age (*p* = 0.183), sex (*p* = 0.218), race/ethnicity (*p* = 0.716), weight (*p* = 0.075), or laterality (*p* = 0.264). There was however a significant difference in operative time between the LIHR and rTAPP groups (20 vs 115 min, *p* < 0.001).

## Discussion

Current evidence does not establish one universally superior approach to adolescent inguinal hernia repair [4, 21]. A nationwide cohort suggests that older adolescents may have a higher risk of recurrence than younger children and young adults [22], whereas a multicenter pediatric cohort found mesh use to be uncommon and recurrence to be low regardless of mesh placement [23]. Within this context, we describe a standardized intraoperative algorithm that selects LIHR or rTAPP according to hernia-specific anatomy. This study introduces a standardized algorithm for selecting a minimally invasive approach to adolescent inguinal hernia repair based on the specific features of each hernia, addressing ongoing variability and lack of consensus in surgical practice. Structured decision-making algorithms have been used across hernia care to promote consistency while incorporating clinical and anatomic factors into treatment selection [24–27]. We found that this algorithm provided a feasible framework for individualized repair, with no recurrences observed during available follow-up. The predominance of rTAPP over LIHR in our cohort likely reflects a referral population of post-pubertal males and underscores the algorithm’s emphasis on intraoperative hernia characteristics.

Contemporary data support the need for a selective rather than uniform approach in adolescents. In a systematic review and meta-analysis, recurrence was low after both mesh and non-mesh repair in adolescents: 1.6% after open non-mesh repair and 1.9% after laparoscopic non-mesh repair, compared with 0.6% after open mesh repair and no reported recurrences after laparoscopic mesh repair [21]. Together with more recent nationwide and multicenter cohorts, these findings do suggest that most adolescents have favorable outcomes after non-mesh repair, while selected older patients or hernias with more complex anatomy may benefit from floor reinforcement [22, 23]. However, we perceive that both pediatric surgeons and pediatric surgery datasets may have blind spots with respect to age. Namely, many surgeons in children’s hospitals refer inguinal hernias in post-pubertal adolescents to adult colleagues, and neither surgeons nor pediatric datasets are aware of young adult recurrences in the years following adolescent repair. We suspect this last point is particularly salient, as we hypothesize that some recurrences may develop gradually through a persistently patulous internal ring after high ligation alone and therefore may not become clinically apparent until patients have left home and are no longer seen in pediatric systems.

Another common defense of high ligation alone is a step-up approach. The reported recurrence rate of up to 6.9% after high ligation in adolescents is higher than rates described in broader pediatric and adult cohorts [13, 28–31]. However, this difference may be acceptable in appropriately selected patients when high ligation provides a shorter, mesh-free operation and does not rule out consideration of a future preperitoneal repair if recurrence occurs. Though true, we favor using available evidence to refine patient selection and use mesh only when recurrence is deemed higher than acceptable in a shared decision-making encounter with patients and families. In our algorithm, LIHR is used for small indirect defects without scrotal extension, whereas rTAPP is favored when diagnostic laparoscopy identifies a direct component, a defect greater than 2 cm, or a long hernia sac with scrotal extension. Most patients in our referred adolescent cohort met one or more of these criteria and underwent rTAPP with mesh reinforcement. Although TAPP may be performed using conventional laparoscopic or robotic techniques, robotic TAPP was the standardized approach used at our institution based on surgeon expertise and platform availability.

Despite the potential benefit of floor reinforcement for selected defects, pediatric surgeons may be reluctant to place mesh in adolescents because of concern for chronic postoperative inguinal pain and other mesh-related morbidity over a long remaining lifespan, as well as uncertainty regarding permanent foreign material in a still-growing patient [4, 21]. In the adolescent systematic review, reported chronic pain ranged from 0 to 11%, although definitions and duration of follow-up varied substantially [21]. The label of chronic pain in studies brings to mind neuropathy with pain, medications, and interventions, but in fact, can be as liberally defined as to include a self-limited paresthesia at 30 days postoperatively. Nevertheless, these concerns support reserving mesh for hernias with specific anatomic features, rather than applying it routinely to all adolescents. Notably, we did not experience any significant mesh complications in this cohort.

An illustration of our algorithm’s individualized application is a 16-year-old male with bilateral inguinal hernias. Diagnostic laparoscopy demonstrated a small right indirect hernia measuring less than 2 cm without a scrotal component and a large left indirect hernia containing omentum with scrotal extension. Following the algorithm, we performed right-sided LIHR and left rTAPP. Applying either approach bilaterally could have exposed the smaller right-sided defect to unnecessary mesh and additional operative time or put the left-sided defect at higher risk of recurrence. This case demonstrates how the algorithm can tailor repair not only to each patient but also to each hernia in the setting of bilateral disease. The minimally invasive approach also permits evaluation of the contralateral groin and identification of occult defects, which can be present in up to 20% of the population [32].

The selection of the optimal minimally invasive surgical approach should be guided by both hernia characteristics and surgeon proficiency, as well as institutional resources [17]. Only surgeons who are proficient in both LIHR and rTAPP should implement this algorithm. This requirement reflects the importance of technical expertise and familiarity with both laparoscopic and robotic platforms in achieving optimal outcomes and minimizing potential complications.

Operative time was substantially longer for rTAPP than for LIHR, reflecting the additional port placement, peritoneal dissection, mesh placement, and intracorporeal suturing required for this approach [5]. This tradeoff should be considered when selecting the operative technique. Our workflow limits unnecessary use of robotic resources by beginning with diagnostic laparoscopy in a robot-equipped operating room and opening and docking the robotic system only when predefined hernia characteristics favor rTAPP. This preserves the procedural efficiency of LIHR for suitable defects while maintaining access to floor reinforcement for selected hernias.

The primary objective of this study is to illustrate the efficacy of tailoring the approach to adolescent minimally invasive inguinal hernia repair. Its retrospective, single-center design and small sample size limit generalizability, and the study was not powered to detect differences in recurrence. Follow-up was also limited, precluding conclusions regarding long-term recurrence, chronic pain, mesh-related morbidity, or patient satisfaction. Prospective internal and external validation in larger cohorts with standardized long-term follow-up is needed to assess the algorithm’s reproducibility and clinical outcomes.

Despite these limitations, our experience demonstrates that a standardized intraoperative algorithm can be implemented to guide individualized minimally invasive inguinal hernia repair in adolescents. By integrating hernia characteristics with surgeon proficiency and institutional resources, the algorithm provides a practical framework for selecting LIHR or rTAPP rather than applying a uniform operative approach to all patients.

## Conclusion

This study contributes to the evolving landscape of pediatric robotic surgery by introducing a standardized algorithm for adolescent inguinal hernia repair. By integrating patient and hernia characteristics with surgical expertise and the robotic platform, this algorithm aims to optimize outcomes and minimize variability in surgical practice. Continued research and collaboration are needed to refine and validate the algorithm, ensuring its applicability and effectiveness.

## Abbreviations

LIHR: Laparoscopic inguinal hernia repair
Rtapp: Robotic-assisted transabdominal preperitoneal hernia repair

## References

[1] Bruns NE, Glenn IC, McNinch NL, Rosen MJ, Ponsky TA (2017) Treatment of routine adolescent inguinal hernia vastly differs between pediatric surgeons and general surgeons. Surg Endosc 31:912–916. 10.1007/s00464-016-5054-z 27357926

[2] Jones VS, Cohen RC (2008) Two decades of minimally invasive pediatric surgery-taking stock. J Pediatr Surg 43:1653–1659. 10.1016/j.jpedsurg.2008.01.006 18779002

[3] Levitt MA, Ferraraccio D, Arbesman MC, Brisseau GF, Caty MG, Glick PL (2002) Variability of inguinal hernia surgical technique: a survey of North American pediatric surgeons. J Pediatr Surg 37:745–751. 10.1053/jpsu.2002.32269 11987092

[4] Lobe TE, Bianco FM (2022) Adolescent inguinal hernia repair: a review of the literature and recommendations for selective management. Hernia 26:831–837. 10.1007/s10029-021-02551-0 35028731

[5] Scrushy MG, Jacobson JC, Pandya SR, Gillory LA (2023) Robotic repair of pediatric hernias: current techniques and practices. Semin Pediatr Surg 32:151261. 10.1016/j.sempedsurg.2023.151261 36736163

[6] Davies DA, Rideout DA, Clarke SA (2020) The International Pediatric Endosurgery Group evidence-based guideline on minimal access approaches to the operative management of inguinal hernia in children. J Laparoendosc Adv Surg Tech A 30:221–227. 10.1089/lap.2016.0453 28140751

[7] Ponsky TA, Nalugo M, Ostlie DJ (2014) Pediatric laparoscopic inguinal hernia repair: a review of the current evidence. J Laparoendosc Adv Surg Tech A 24:183–187. 10.1089/lap.2014.9998 24625349

[8] Dreuning K, Maat S, Twisk J, van Heurn E, Derikx J (2019) Laparoscopic versus open pediatric inguinal hernia repair: state-of-the-art comparison and future perspectives from a meta-analysis. Surg Endosc 33:3177–3191. 10.1007/s00464-019-06960-2 PMC6722044 31317333

[9] Ramser M, Baur J, Keller N, Kukleta JF, Dörfer J, Wiegering A, Eisner L, Dietz UA (2021) Robotic hernia surgery I. English version: robotic inguinal hernia repair (r-TAPP). Video report and results of a series of 302 hernia operations. Chirurg 92(Suppl 1):1–13. 10.1007/s00104-021-01446-1 PMC8695418 34928424

[10] Kozlov Y, Kapuller V (2022) Results of using the method of full extraperitoneal endoscopically assisted ligation of the hernial sac with inguinal hernia in children. J Pediatr Surg 57:153–157. 10.1016/j.jpedsurg.2021.09.026 34674845

[11] Chu CB, Chen J, Shen YM, Liu SJ, Sun L, Nie YS, Liu J, Cao JX, Du HD, Zou ZY, Yuan X (2020) Individualized treatment of pediatric inguinal hernia reduces adolescent recurrence rate: an analysis of 3006 cases. Surg Today 50:499–508. 10.1007/s00595-019-01940-4 31858238

[12] Fan G, Yao G (2023) Analysis of the efficacy of laparoscopic high hernia sac ligation in adolescent indirect hernia. BMC Surg 23:160. 10.1186/s12893-023-02048-w PMC10265881 37312075

[13] Taylor MA, Cutshall ZA, Eldredge RS, Kastenberg ZJ, Russell KW (2021) High ligation in adolescents: is it enough? J Pediatr Surg 56:1865–1869. 10.1016/j.jpedsurg.2020.11.006 33234290

[14] Denning NL, Kallis MP, Prince JM (2020) Pediatric robotic surgery. Surg Clin North Am 100:431–443. 10.1016/j.suc.2019.12.004 32169188

[15] Jacobson JC, Pandya SR (2023) Pediatric robotic surgery: an overview. Semin Pediatr Surg 32:151255. 10.1016/j.sempedsurg.2023.151255 36736161

[16] Mattioli G, Pini Prato A, Razore B, Leonelli L, Pio L, Avanzini S, Boscarelli A, Barabino P, Disma NM, Zanaboni C, Garzi A, Martigli SP, Buffi NM, Rosati U, Petralia P (2017) Da Vinci robotic surgery in a pediatric hospital. J Laparoendosc Adv Surg Tech A 27:539–545. 10.1089/lap.2016.0390 28278402

[17] Ram C, Pitt JB, Wallace MW, Goldstein SD, Zamora IJ (2024) Framework for pediatric robotic surgery program development. Semin Pediatr Surg 33:151389. 10.1016/j.sempedsurg.2024.151389 38245993

[18] van Haasteren G, Levine S, Hayes W (2009) Pediatric robotic surgery: early assessment. Pediatrics 124:1642–1649. 10.1542/peds.2008-3822 19917586

[19] Hey MT, Mayhew MM, Rico S, Calisto J, Alkhoury F (2021) Initial experience with robotic inguinal hernia repair in the adolescent population. J Laparoendosc Adv Surg Tech A 31:1346–1350. 10.1089/lap.2021.0301 34252321

[20] Waite KE, Herman MA, Doyle PJ (2016) Comparison of robotic versus laparoscopic transabdominal preperitoneal (TAPP) inguinal hernia repair. J Robot Surg 10:239–244. 10.1007/s11701-016-0580-1 27112781

[21] Reistrup H, Andresen K, Rosenberg J (2023) Low incidence of recurrence and chronic pain after groin hernia repair in adolescents: a systematic review and meta-analysis. Langenbecks Arch Surg 408:211. 10.1007/s00423-023-02947-9 PMC10220125 37233839

[22] Sincavage J, Sullivan GA, Fritsch A, Palmisano Z, Raval MV, Blakely M, Shah AN, Gulack BC (2025) Older children undergoing inguinal hernia repair have higher recurrence rates than younger children and adults: a nationwide cohort study. J Pediatr Surg 60:162083. 10.1016/j.jpedsurg.2024.162083 39693824

[23] Heller K, Ochoa BV, Eldredge RS, Padilla BE (2026) Evaluation of mesh placement in adolescents undergoing inguinal hernia repair: a multicenter retrospective cohort study. J Am Coll Surg 242:705–711. 10.1097/XCS.0000000000001685 41195884

[24] Pawlak M, East B, de Beaux AC (2021) Algorithm for management of an incarcerated inguinal hernia in the emergency settings with manual reduction. Taxis, the technique and its safety. Hernia 25:1253–1258. 10.1007/s10029-021-02429-1 PMC8147903 34036484

[25] Lange JFM, Kaufmann R, Wijsmuller AR, Pierie JPEN, Ploeg RJ, Chen DC, Amid PK (2015) An international consensus algorithm for management of chronic postoperative inguinal pain. Hernia 19:33–43. 10.1007/s10029-014-1292-y 25138620

[26] Baig SJ, Priya P (2021) Management of ventral hernia in patients with BMI > 30 kg/m2: outcomes based on an institutional algorithm. Hernia 25:689–699. 10.1007/s10029-020-02318-z 33044608

[27] Shehata SM, Attia MA, El Attar AA, Ebid AE, Shalaby MM, ElBatarny AM (2018) Algorithm of laparoscopic technique in pediatric inguinal hernia: results from experience of 10 years. J Laparoendosc Adv Surg Tech A 28:755–759. 10.1089/lap.2017.0273 29424624

[28] Chen K, Xiang G, Wang H, Xiao F (2011) Towards a near-zero recurrence rate in laparoscopic inguinal hernia repair for pediatric patients. J Laparoendosc Adv Surg Tech A 21:445–448. 10.1089/lap.2009.0294 21235425

[29] Ein SH, Njere I, Ein A (2006) Six thousand three hundred sixty-one pediatric inguinal hernias: a 35-year review. J Pediatr Surg 41:980–986. 10.1016/j.jpedsurg.2006.01.020 16677897

[30] Lee CS, Kim JH, Choi BJ, Lee JI, Lee SC, Lee YS, Oh ST, Kim HJ (2020) Retrospective study on prevalence of recurrent inguinal hernia: a large-scale multi-institutional study. Ann Surg Treat Res 98:51–55. 10.4174/astr.2020.98.1.51 PMC6940424 31909050

[31] Ramsay G, Scott NW, Jansen JO (2020) A 19 year population-based cohort study analysing reoperation for recurrence following laparoscopic and open inguinal hernia repairs. Hernia 24:793–800. 10.1007/s10029-019-02073-w PMC7395908 31786699

[32] Jarrard JA, Arroyo MR, Moore BT (2019) Occult contralateral inguinal hernias: what is their true incidence and should they be repaired? Surg Endosc 33:2456–2458. 10.1007/s00464-018-6528-y PMC6647449 30327914

